# Analyzing the phylogeny of poplars based on molecular data

**DOI:** 10.1371/journal.pone.0206998

**Published:** 2018-11-09

**Authors:** An-Pei Zhou, Dan Zong, Pei-Hua Gan, Xin-Lian Zou, Yao Zhang, Li Dan, Cheng-Zhong He

**Affiliations:** 1 Key Laboratory for Forest Genetic and Tree Improvement and Propagation in Universities of Yunnan Province, Southwest Forestry University, Kunming, Yunnan, China; 2 Key Laboratory of Biodiversity Conservation in Southwest China, State Forestry Administration, Southwest Forestry University, Kunming, Yunnan, China; 3 Yunnan Academy of Biodiversity, Southwest Forestry University, Kunming, Yunnan, China; 4 Key Laboratory for Forest Resources Conservation and Utilization in the Southwest Mountains of China, Ministry of Education, Southwest Forestry University, Kunming, Yunnan, China; National Cheng Kung University, TAIWAN

## Abstract

Methods for constructing trees using DNA sequences, known as molecular phylogenetics, have been applied to analyses of phylogenetic origin, evolutionary relatedness and taxonomic classification. Combining data sequenced in this study and downloaded from GenBank, we sampled 112 (chloroplast data) / 122 (*ITS* data) specimens belonging to 49 (chloroplast data) / 46 (*ITS* data) poplar species or hybrids from six (chloroplast data) / five sections (*ITS* data). Maximum parsimony and Bayesian inference were used to analyze phylogenetic relationships within the genus *Populus* based on eight chloroplast combinations and *ITS* regions. The results suggested that Bayesian inference might be more suitable for the phylogenetic reconstruction of *Populus*. All *Populus* species could be divided into two clades: clade 1, including subclades 1 and 2, and clade 2, including subclades 3 and 4. Species within clade 1, involving five sections except for *Leuce*, clustered coinciding with their two specific main geographical distribution areas: China (subclade 1) and North America (subclade 2). Clustering in subclade 3, section *Leuce* was confirmed to be of monophyletic origin and independent evolution. Its two subsections, namely *Albidae* and *Trepidae*, could be separated by chloroplast data but had frequent gene flow based on *ITS* data. Phylogeny analysis based on chloroplast data demonstrated once more that section *Aigeiros* was paraphyletic and further showed that the *P*. *deltoides* lineage is restricted in subclade 2 and that *P*. *nigra* lineage, located in subclade 3, originated from a hybrid of which an *Albidae* ancestor species was the material parent. Similarly, section *Tacamahaca* was found to be paraphyletic and had two lineages: a clade 1 lineage, such as *P*. *cathayana*, and a clade 2 lineage, such as *P*. *simonii*. Section *Leucoides* was paraphyletic and closely linked to section *Tacamahaca*. Their section boundaries were not conclusively delimitated by sequencing information.

## Introduction

Poplars (*Populus* L.), one of the world’s most important forest trees, are accepted as model trees due to their high growth, strong adaptability, easy propagation and small genome[1–3]. In plant systematic databases, Eckenwalder classified *Populus* into 22 species in six sections: *Tacamahaca*, *Aigeiros*, *Leuce*, *Leucoides*, *Turanga* and *Abaso*[4], but the Flora of China recorded 71 species from five sections (except for *Abaso*)[5]. They are widely distributed across the northern hemisphere[5–6]. China has rich poplar germplasm resources because it is one of the most important poplar distribution areas. In Flora of China, 47 species are endemic and relatively unknown outside the country[4–5, 7]. Many studies have only investigated a few species, such as *P*. *tomentosa* and *P*. *euphratica*, while almost completely ignoring other species. To develop and utilize resources as an important foundation for basic and applied research, it is imperative to understand the genetic and phylogenetic relationships among *Populus*.

Frequent natural interspecific hybridization in *Populus* has resulted in difficulty with its classification. Most studies[4, 8–11] have concluded that the genus *Populus* is monophyletic, but the taxonomic and phylogenetic relationships within this genus are ambiguous. Molecular evidence has shown differing results. Among the five sections, *Turanga* is controversial. AFLP[12] and single-copy nuclear DNA[11] data suggest that section *Turanga* forms a separate clade, while chloroplast data show that this section is related to section *Tacamahaca*[11]. Section *Leuce* is thought to be monophyletic[9–12], and the main arguments are whether it is the basal lineage and how to assess the taxonomic positions of its two subsections (*Albidae* and *Trepidae*). Most studies have demonstrated a close relationship between sections *Tacamahaca* and *Aigeiros*[9–12]. However, both sections have relatively high intrasectional differences and are likely polyphyletic[11]. This has led to a lack of information, such as the number of subsections (or lineages) and the relationship between subsections (or lineages). Section *Leucoides* is often ignored and has been rarely studied. Wang *et al*.[11] showed that section *Leucoides* was related to sections *Tacamahaca* and *Aigeiros*. Cervera *et al*.[12] suggested that *P*. *lasiocarpa* and *P*. *violascens* of section *Leucoides* were classified into section *Tacamahaca*. Meanwhile, the classification of some endemic species as separate species is still doubtful. For example, *P*. *gonggaensis* has annual shoots with a thin pubescence and is subtly different from species that have dense, crimped villi or are hairless, such as *P*. *lasiocarpa*. This variation is difficult to morphologically identify by eye.

The highly different evolution rates among DNA regions can infer the phylogenetic relationships between *Populus* species at any classification level. Ideally, the evolutionary relationships among species are unique and can be described using a species tree. However, we have only explored these relationships using gene trees based on one or a few gene regions[13–15]. With increasing amounts of molecular evidence becoming available, an increasing number of cases showing inconsistencies among gene trees have been found, such as the placement of *P*. *nigra*, which showed high genetic differentiation with consectional *P*. *deltoides* when *Populus* was molecularly analyzed[9, 11, 16–17].

Conflicting gene trees were easily obtained from the chloroplast and mitochondrial genomes with uniparental inheritance and from nuclear genomes with biparental inheritance[18–19]. The chloroplast genome is associated with organism phylogeny but is not hybrid and allopolyploid in nature. The nuclear genome can be used to analyze hybrid origin and reticulate evolution but does not confirm whether the gene tree is attributable to paralogous copies[20–21]. Therefore, combining gene trees created using chloroplast and nuclear datasets is imperative if we are to improve understanding of the phylogenetic relationships among *Populus*.

In this study, we selected eight chloroplast regions, including four coding (*rbcL-a*, *matK*, *rpoB* and *ropC1*)[22–24] and four noncoding (*psbA-trnH*, *psbI-psbK*, *atpF-atpH* and *trnL-F*) regions[25–27], together with one nuclear ribosomal internal transcribed spacer (*ITS*)[28]. We analyzed and compared phylogenetic information from the chloroplast and nuclear genomes and focused on revealing the intrasectional relationships and reticulate evolution of *Populus*.

## Materials and methods

### Taxon sampling

We collected leaves from *Populus* species across China and obtained sequence data from GenBank. *Salix matsudana* was used as an outgroup. The information on sampled species and locations is shown in S1 Table. No permits were required for the described study because Chinese legislation does not forbid access to study poplar in nature reserves and national parks. We confirm that the study specimens included only Salicaceae samples, and these samples were not involve from endangered or protected species.

A total of 112 specimens, representing 49 species or hybrids from six sections, were used for chloroplast DNA phylogeny, in which 80 specimens we collected were successfully amplified and sequenced for chloroplast regions. To avoid stochastic error, we only downloaded chloroplast genome sequences from *Populus* and extracted and combined regions. Of 122 specimens representing 46 species or hybrids from five sections provided for *ITS* phylogeny, 62 of the specimens we collected were successfully sequenced for regions.

### DNA extraction, PCR amplification and sequencing

*Populus* leaves were dried in silica gel, and modified SDS[29] was used for genomic DNA extraction. The 25-μl PCR amplification reaction, containing 1 μl of DNA (approximately 20 ng), 12.5 μl of 2× Taq MasterMix, 1 μl of both reverse and forward primers (10 pmol) and 9.5 μl of ddH2O, was performed as follows: one cycle of initial denaturation at 94°C for 4 min; 35 cycles of denaturation at 94°C for 30 s, annealing at approximately 54°C–59°C (depending upon the primer sets used) for 45 s, and elongation at 72°C for 60 s; and a final cycle of elongation for 5 min. Then, sequencing was completed by Sangon Biotech Co., Ltd. (Shanghai, China).

### Data analysis

After the DNA sequences had been edited and aligned using Clustal X 2.0[30], MEGA 5.02[31] was used to measure the sequence lengths and count the number of variable and informative sites for each region. Pairwise distances were calculated on the basis of the Kimura 2-parameter (K2P) model using MEGA 5.02 with the pairwise deletion and uniform rates options.

The incongruence length difference (ILD) test[32] was used to evaluate the congruence of eight chloroplast regions in PAUP*4.0b10[33]. The *ITS* data and the combined data for all eight chloroplast regions, with *Salix matsudana* as the outgroup, allowed us to carry out a phylogenic analysis of the genetic relationships between *Populus* species using two algorithms. Maximum parsimony analysis was undertaken. A full heuristic search was used for branch support with 1000 replicates[34]. Bayesian inference was performed in MrBayes 3.1.2[35] based on a best-fitting nucleotide substitution model and the Akaike information criterion (AIC) derived from Modeltest 3.7[36]. Parameter settings included 1,000,000 generations, in which trees were sampled once every 100 generations and the first 25% of sampled trees were calculated as burn-in. Posterior probability was also estimated using Markov chain Monte Carlo (MCMC).

## Results

### Length and number of variable and informative sites in each region

The high congruence for all eight chloroplast regions was identified by the ILD test (*P* = 0.18 > 0.05). As shown in Table 1, the combined region had 188 variable sites and 84 informative sites and was 5171 bp long. *ITS* region analysis suggested that this region contained 74 informative and 92 variable sites that belonged to a 575-bp aligned sequence.

**Table 1:** Length and number of variable and informative sites identified in the combined chloroplast and *ITS* regions.

Region	Aligned sequence length / bp	No. variable sites	No. informative sites
Eight chloroplast regions	5171	188	84
*ITS*	575	92	74

### Pairwise distance analysis

The average K2P distances (Table 2) based on the combined eight chloroplast and the *ITS* regions in *Populus* were 0.00292 and 0.01818, respectively. The chloroplast combination dataset showed that the pairwise distance between the six sections ranged from 0.00211 (*Abaso* and *Aigeiros*) to 0.00397 (*Leuce* and *Abaso*), while it ranged from 0.01057 (*Tacamahaca* and *Leucoides*) to 0.03754 (*Leuce* and *Turanga*) by *ITS* analysis.

**Table 2:** Pairwise distances, based on K2P distance, between the different *Populus* sections.

	*Tacamahaca*	*Aigeiros*	*Leuce*	*Leucoides*	*Turanga*	*Abaso*
*Tacamahaca*	****	0.01450	0.02442	0.01057	0.02927	NA
*Aigeiros*	0.00320	****	0.02189	0.01099	0.02303	NA
*Leuce*	0.00306	0.00313	****	0.01970	0.03754	NA
*Leucoides*	0.00216	0.00322	0.00318	****	0.02490	NA
*Turanga*	0.00301	0.00364	0.00364	0.00280	****	NA
*Abaso*	0.00339	0.00211	0.00397	0.00323	0.00341	****

Pairwise distances calculated using the chloroplast combination data are showed below the diagonal, and those calculated using the *ITS* data are shown above the diagonal. “NA” indicates absent data.

**Fig 1:**
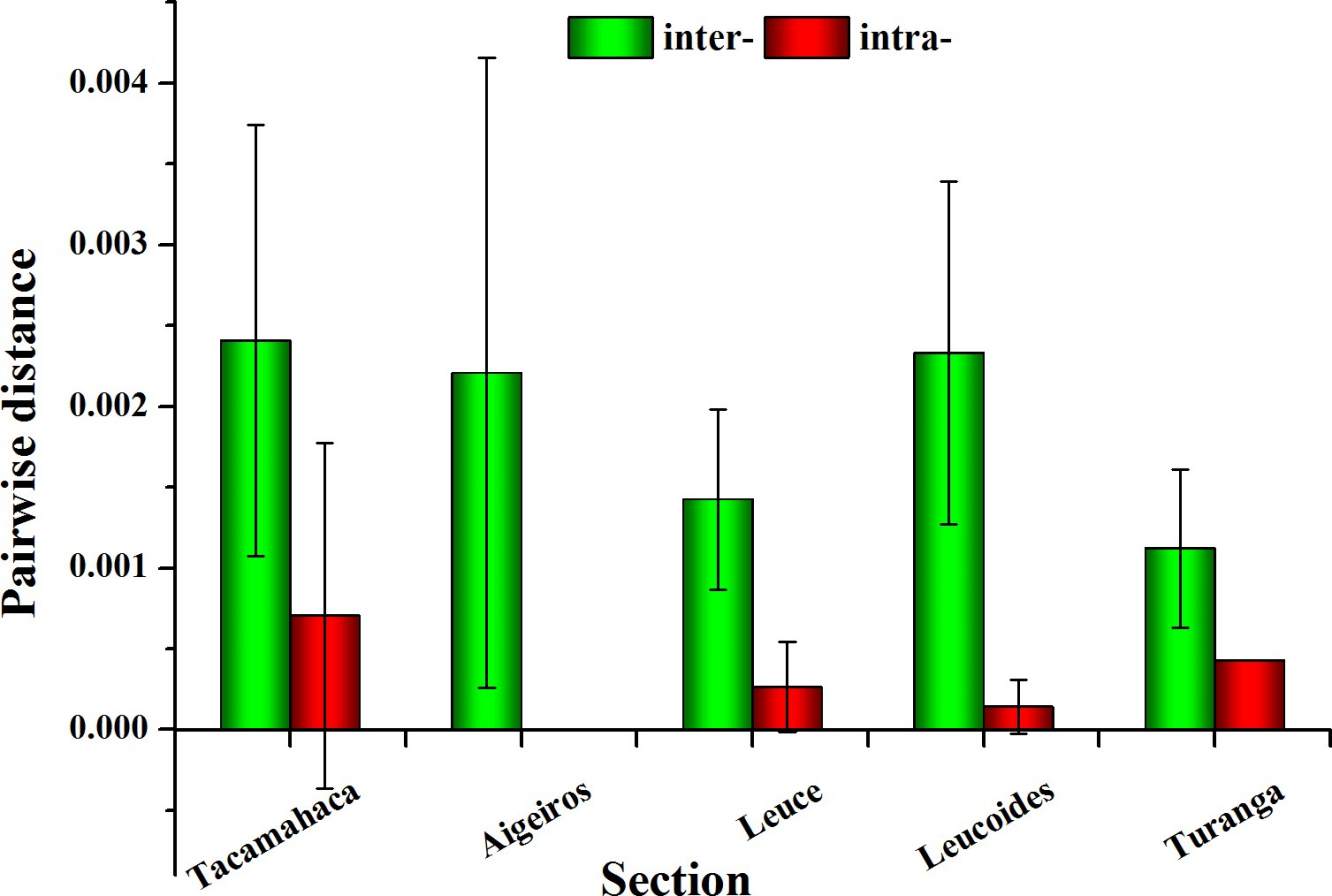
Average inter- and intraspecific pairwise distances for each section using the chloroplast combination data.

**Fig 2:**
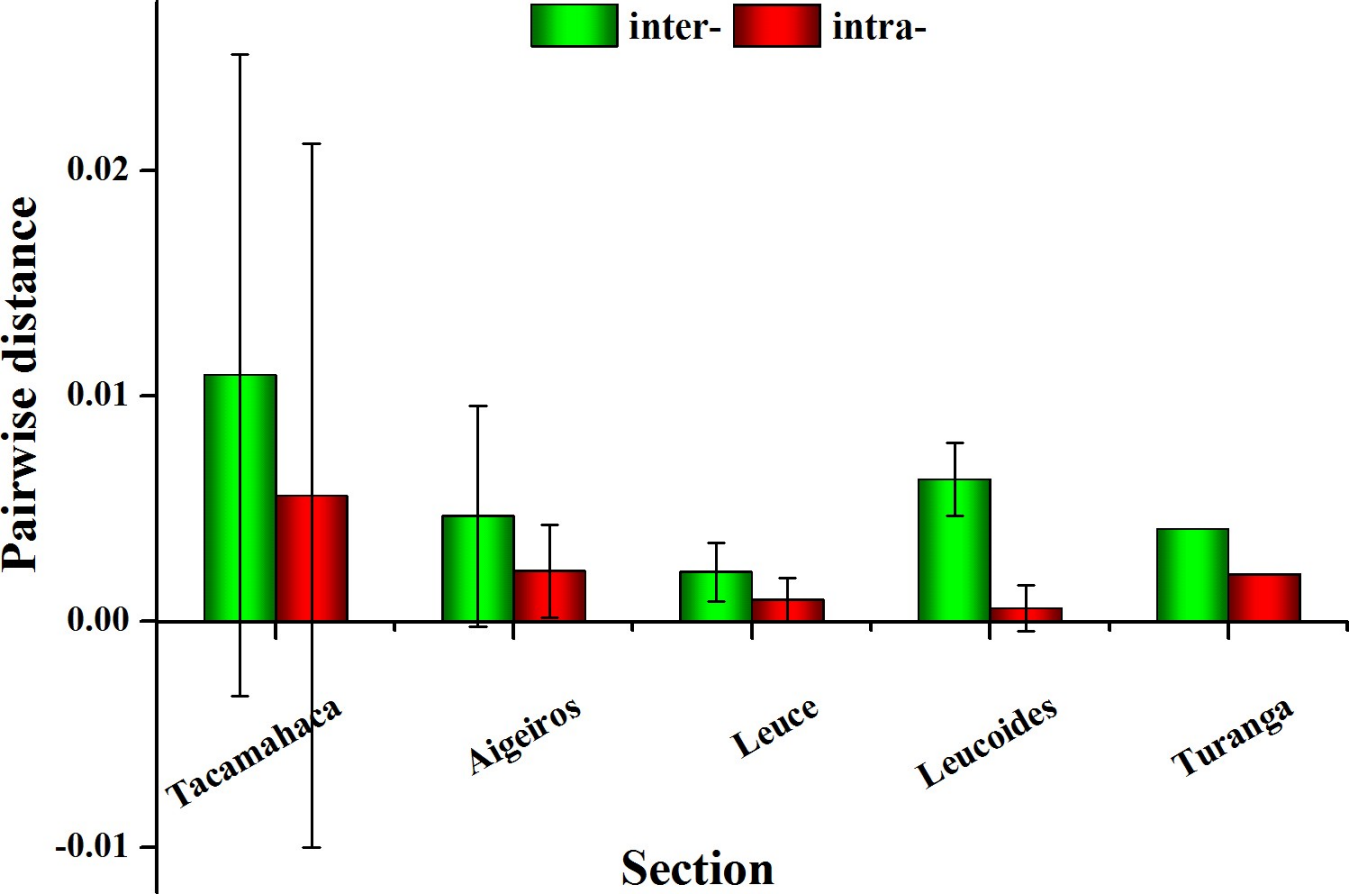
Average inter- and intraspecific pairwise distances for each section using the *ITS* data.

The chloroplast combination dataset showed that the average interspecific pairwise distance was highest for section *Tacamahaca* at 0.00241, followed by *Leucoides* at 0.00233, *Aigeiros* at 0.00221, *Leuce* at 0.00142 and *Turanga* at 0.00112. The rank order for average intraspecific divergence was *Tacamahaca* (0.00071) > *Turanga* (0.00043) > *Leuce* (0.00026) > *Leucoides* (0.00014) > *Aigeiros* (0). The *ITS* region dataset showed that the highest inter- and intraspecific distances were in both sections *Tacamahaca* (0.01093 and 0.00558, respectively), and the lowest values were present in sections *Leuce* (0.00221) and *Leucoides* (0.00059). The error bars for pairwise distances are the standard deviations of linear fit. As shown in Figs 1 and 2, large error bars between consectional species were observed for sections *Tacamahaca*, *Aigeiros* and *Leucoides*, which suggested that the fluctuation of interspecific variation within section was high.

### Phylogenetic analysis

**Fig 3:**
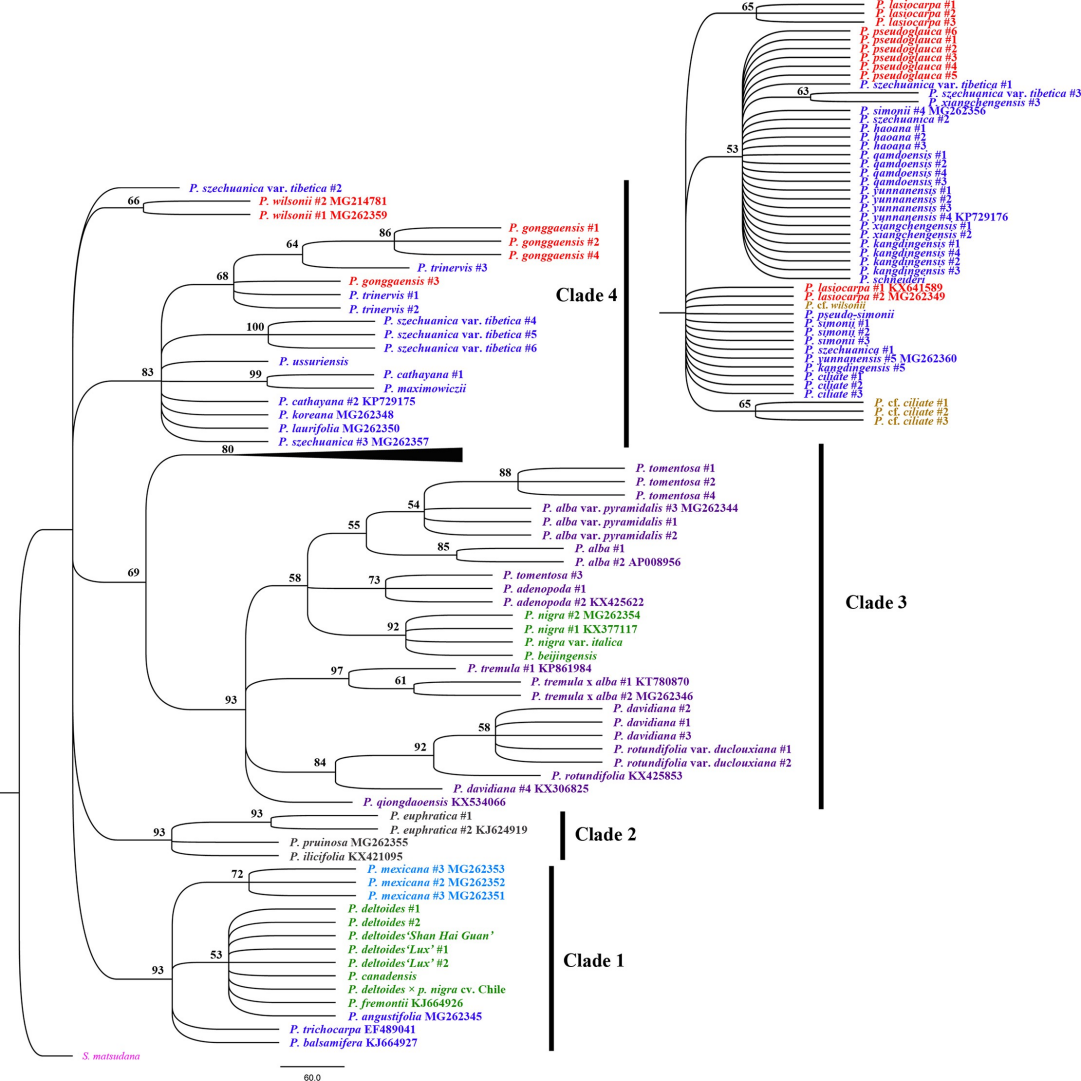
MP phylogenetic tree based on the combined data for all eight chloroplast regions. Bootstrap support values are reported for nodes over 50%. The traditional species taxa, based on morphological characteristics, are shown in different colors. The outgroup is pink, *Tacamahaca* is blue, *Aigeiros* is green, *Leuce* is purple, *Leucoides* is red, *Turanga* is brown, *Abaso* is skyblue, and hybrids are yellow.

**Fig 4:**
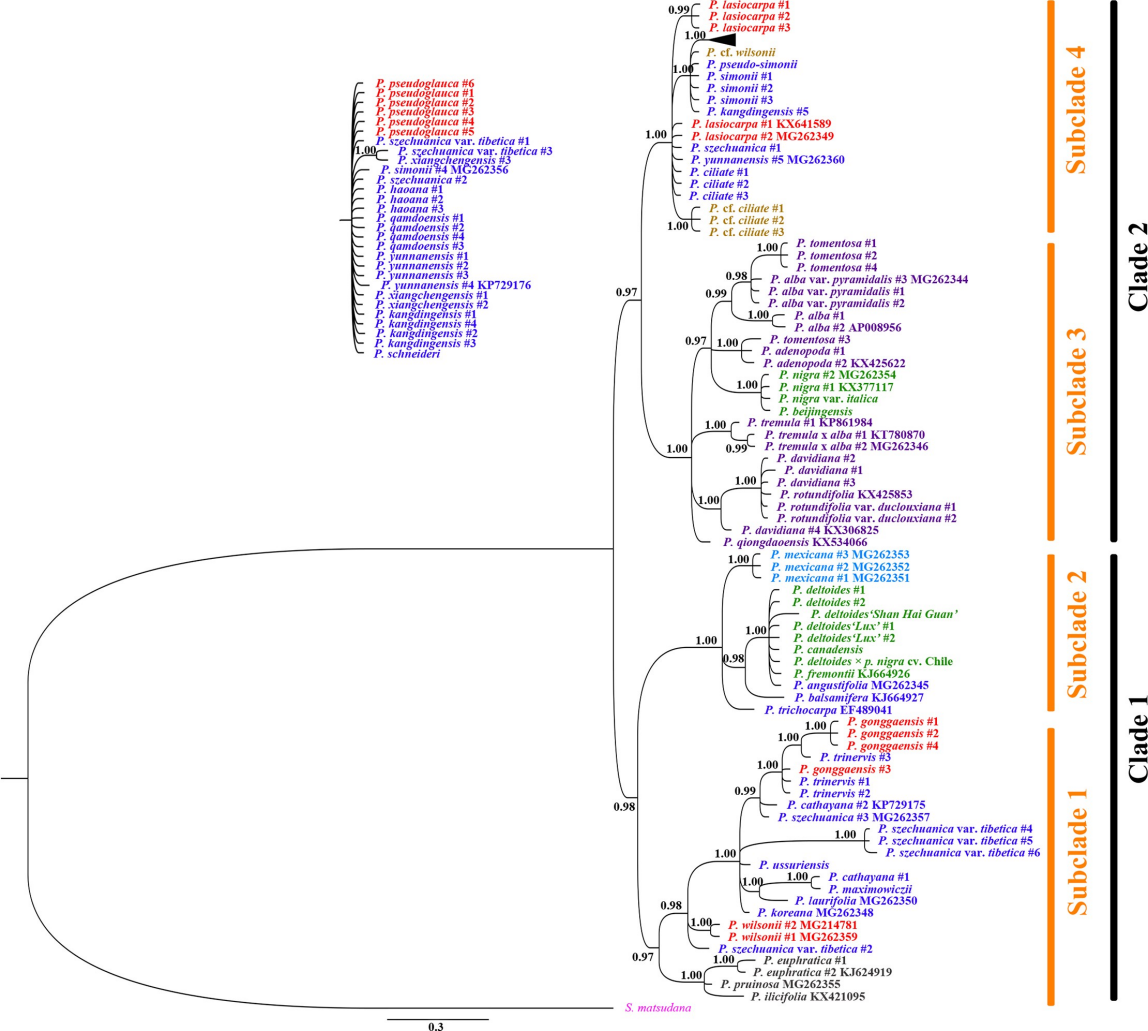
Bayes phylogenetic tree based on the combined data from all eight chloroplast regions. The *K81uf+I+G* model for appropriate nucleotide substitution was constructed using Modeltest 3.7 with the AIC. Bayesian posterior probability values are reported for nodes over 50%. The traditional species taxa, based on morphological characteristics, are shown in different colors. The outgroup is pink, *Tacamahaca* is blue, *Aigeiros* is green, *Leuce* is purple, *Leucoides* is red, *Turanga* is brown, *Abaso* is skyblue, and hybrids are yellow.

Using *S*. *matsudana* as the outgroup, the phylogenetic relationships among *Populus*, based on the chloroplast combination dataset, showed that four major clades with high or moderate support values were distinguished in the MP tree (Fig 3). Bayesian inference further adjusts this distribution with high posterior probabilities (≥ 0.97) and divided all *Populus* specimens into four subclades belonging to two clades (Fig 4). Subclade 1 (0.97 posterior probabilities) contained all species in section *Turanga* and partial specimens in sections *Tacamahaca and Leucoide*, such as *P*. *ussuriensis*, *P*. *maximowiczii*, *P*. *cathayana*, *P*. *trinervis*, *P*. *laurifolia*, *P*. *koreana*, *P*. *gonggaensis* and *P*. *wilsonii*. In this subclade, sections *Tacamahaca and Leucoide* were mixed (0.98 posterior probabilities), and they were clearly separated from section *Turanga* (1.00 posterior probabilities). All American *Populus* species formed subclade 2 (1.00 posterior probabilities), in which *P*. *mexicana* was identified with 1.00 posterior probabilities. These two subclades constitute clade 1, with 0.98 posterior probabilities. Subclade 3 included all species from section *Leuce*, together with *P*. *nigra*, *P*. *nigra* var. *italica* and *P*. *beijingensis* in section *Aigeiros* (1.00 posterior probabilities). Clade 4, with 88% bootstrap support, consisted of *P*. *lasiocarpa* and *P*. *pseudoglauca* in section *Leucoides* and some specimens (except for those found in clade 1) in section *Tacamahaca*.

**Fig 5:**
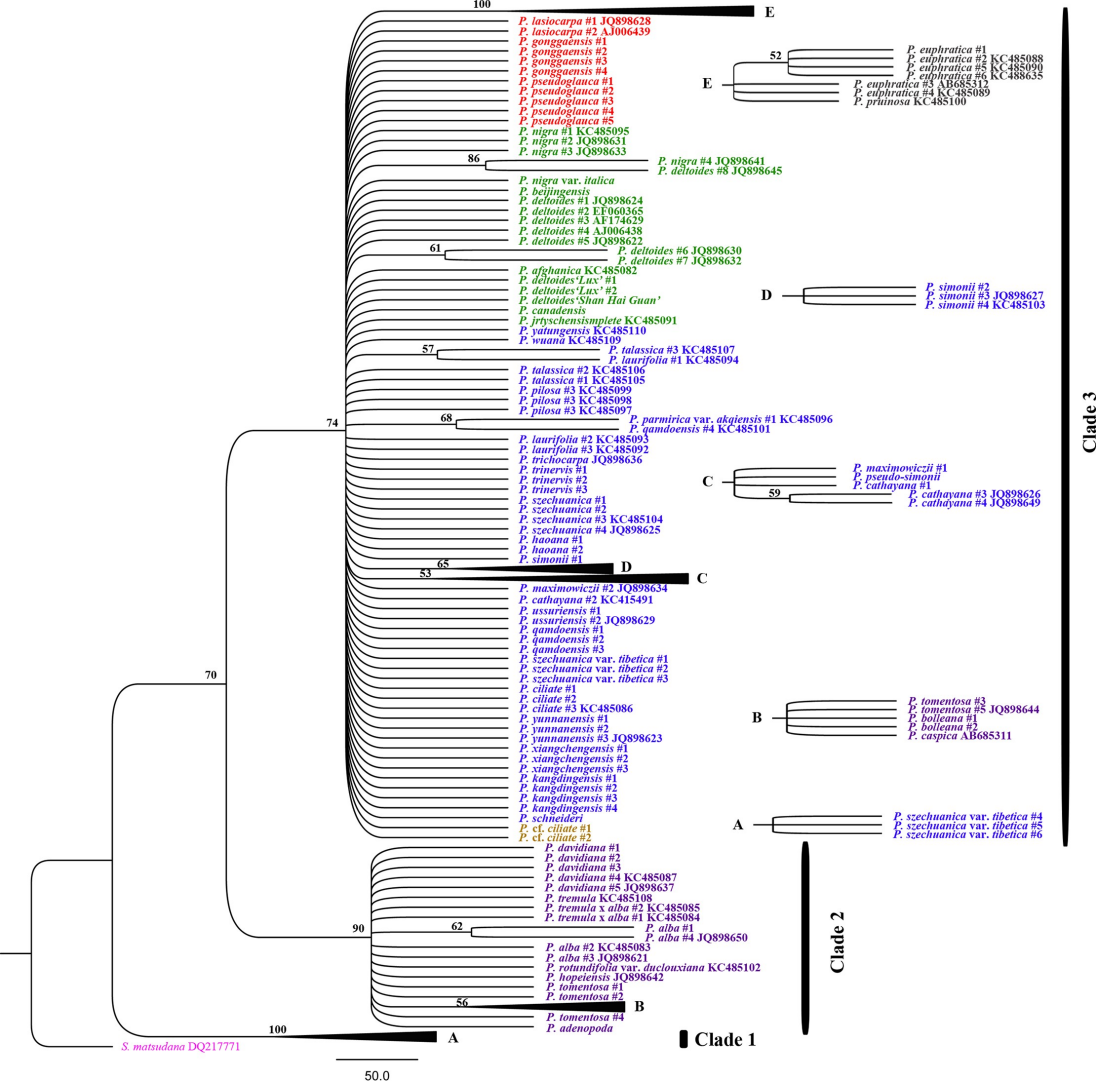
MP phylogenetic tree based on *ITS* regions. Bootstrap support values are reported for nodes over 50%. The traditional species taxa, based on morphological characteristics, are shown in different colors. The outgroup is pink, *Tacamahaca* is blue, *Aigeiros* is green, *Leuce* is purple, *Leucoides* is red, *Turanga* is brown, and hybrids are yellow.

**Fig 6:**
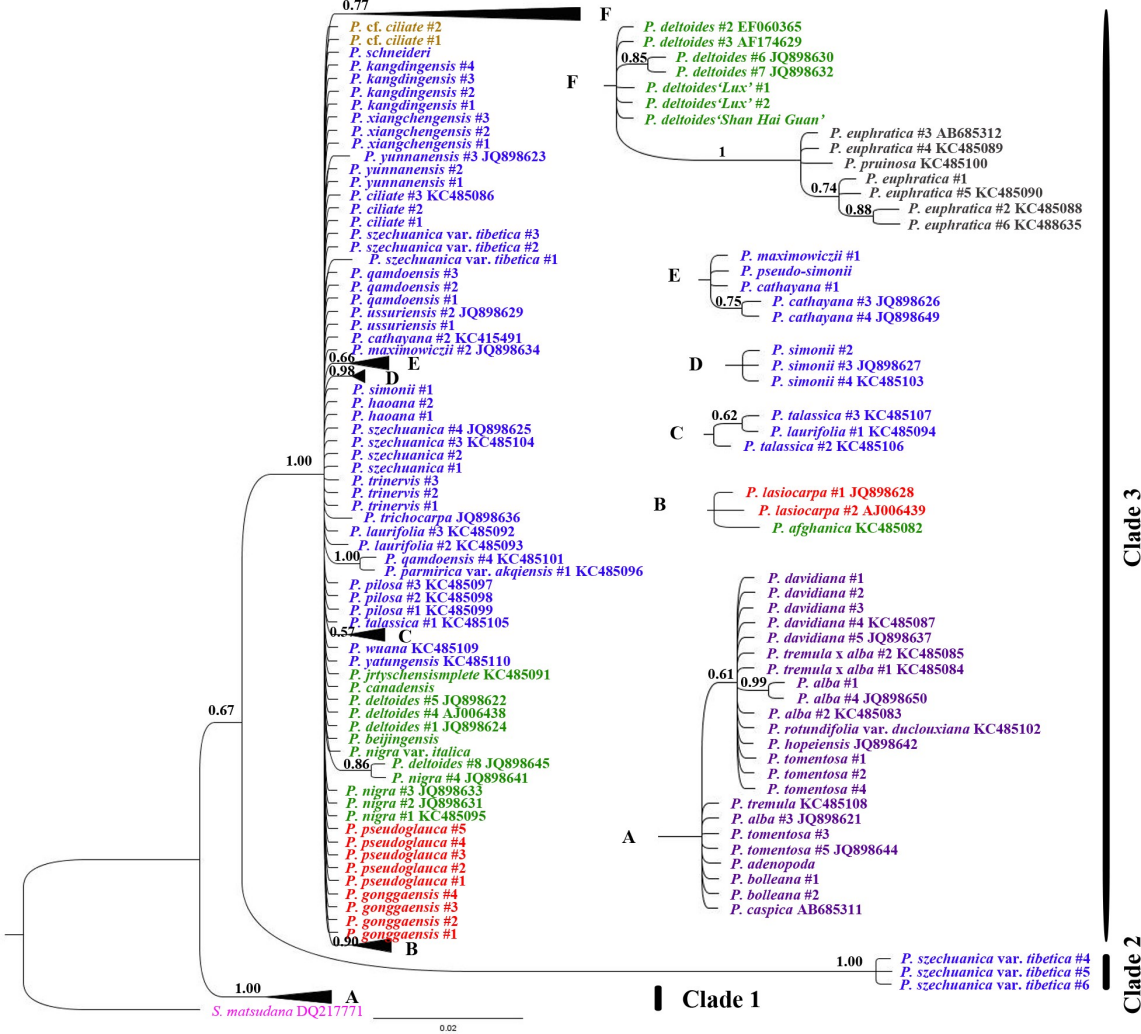
Bayes phylogenetic tree based on *ITS* regions. The *TVM+I+G* model for appropriate nucleotide substitution was constructed using Modeltest 3.7 with the AIC. Bayesian posterior probability values are reported for nodes over 50%. The traditional species taxa, based on morphological characteristics, are shown in different colors. The outgroup is pink, *Tacamahaca* is blue, *Aigeiros* is green, *Leuce* is purple, *Leucoides* is red, *Turanga* is brown, and hybrids are yellow.

The MP tree (Fig 5) for the *Populus* phylogeny based on the *ITS* data showed that three clades were identified. *P*. *szechuanica* var. *tibetica* from Tibet was independent of the other *Populus* species (100% bootstrap support) and was located at the base of tree as clade 1. All species from section *Leuce* were clustered in clade 2, with 90% bootstrap support, and they were clearly separated from the others. Sections *Tacamahaca*, *Aigeiros*, *Leucoides* and *Turanga* and their natural hybrids were sister taxa (74% bootstrap support) in clade 3, in which *Turanga* could be identified with 100% bootstrap support. Compared with the above MP analysis, Bayesian inference (Fig 6) more strongly supported these three clades, with 1.00 posterior probabilities. Bayesian tree analysis also more clearly showed the phylogenetic relationships of some specimens from sections *Tacamahaca*, *Aigeiros*, *Leucoides* and *Turanga*. For instance, *P*. *afghanica* in section *Aigeiros* was clustered with *P*. *lasiocarpa* in section *Leucoides*, which was supported with 0.90 posterior probabilities. However, a number of specimens were characterized with “comb” and could not be identified. Some specimens belonging one species were separated, such as *P*. *simonii*, or were clustered with specimens of other species, such as *P*. *qamdoensis*.

## Discussion

### The incongruence gene trees for *Populus*

One of most notable difficulties in phylogenetic reconstruction is the widespread occurrence of incongruence among methods and among individual genes or different genomic regions[37–38]. Incongruence among methods and genes[37] has been generally accepted and is also shown in the phylogeny results produced by this study. High incongruence was associated with differences between chloroplast and *ITS* regions due to actual differences in their evolutionary histories. High frequency hybridization events played important roles in *Populus* phylogenies, which is reflected by *ITS* tree analysis. Four section species (except for *Leuce*) were not clearly separated and formed a “comb” clade. Comprising similar numbers of informative sites as in the *ITS* analysis (Table 1), combined chloroplast regions effectively discriminated clustering of section, subsection and similar species with high reliability.

Differences in the gene trees based on the same data were attributed to differences between algorithms models. In comparison, we found that the support values from Bayesian posterior probabilities were higher than those from maximum parsimony. Furthermore, the Bayes trees were able to group together relative species because of the high posterior probabilities derived from calculating the statistical likelihood of their sequences. For instance, *P*. *wilsonii* and *P*. *szechuanica* var. *tibetica* #2 were independent of four clades in the chloroplast MP tree, but they were clustered into subclade 1 of the Bayes tree with high posterior probabilities. Clade 3 in the chloroplast MP tree was only identified with 69% bootstrap support, but it was supported with 0.97 posterior probabilities as clade 2 in the Bayes tree. Therefore, Bayesian inference is more suitable for phylogenetic reconstructions of *Populus*.

### The phylogenetic relationships between subsections

Section *Leuce* has been classified into two subsections: *Albidae* and *Trepidae*[12,39–41], which however are not clearly separated by nuclear data[9,11,41], chloroplast data[9,11,42], RAPD data[43] or AFLP data[44]. The major disagreement centers around the taxonomic position of *P*. *adenopoda*. In this study, we sampled 13 species or hybrids from section *Leuce*: *P*. *tomentosa*, *P*. *alba* var. *pyramidalis*, *P*. *caspica* and *P*. *alba* represented subsection *Albidae*, and *P*. *rotundifolia*, *P*. *tremula*, *P*. *davidiana*, *P*. *hopeiensis*, *P*. *qiongdaoensis* and *P*. *adenopoda* represented subsection *Trepidae*. The chloroplast phylogenetic trees showed that subsection *Albidae* could be identified and its species grouped with *P*. *adenopoda*. *P*. *rotundifolia*, *P*. *rotundifolia* var. *duclouxiana* and *P*. *davidiana* could not be clearly separated, and they are sister to *P*. *tremula*, *P*. *qiongdaoensis* and species in subsection *Albidae*. The *ITS* phylogenetic trees showed that the two subsections could not be clearly separated. These results suggested that subsection *Albidae* was monophyletic and that there was frequent gene flow between the two subsectional species, especially for *P*. *adenopoda*.

The section *Aigeiros* is composed of two main species, *P*. *nigra* and *P*. *deltoides*. Some molecular evidence has shown significant genetic differences between these species[11–12, 45–48]. ISSR analysis supported the suggestion that *P*. *nigra* grouped with species in section *Tacamahaca*[10], whereas AFLP analyses suggested that *P*. *deltoides* grouped with *Tacamahaca* species[12]. The study of Li *et al*.[44] was able to divide these two species using AFLP markers, but they were still in one clade. Chloroplast data[9,11,49] showed that *P*. *nigra* grouped with species in section *Leuce*, which suggested a possible hybrid origin for *P*. *nigra* after comparing the nuclear sequences[9, 11].

We agree with the opinion that *P*. *nigra* is a hybrid derived from a natural cross between section *Leuce* as the maternal parent and *P*. *deltoides* as the paternal parent. Furthermore, subsection *Albidae* is highly likely to be the maternal parent because the *Albidae* and *P*. *nigra* lineages share a common ancestor. *P*. *nigra* var. *italica* and *P*. *beijingensis* (*P*. *nigra* var. *italica* × *P*. *cathayana*) belong to the maternal lineage of *P*. *nigra*. Chloroplast data showed that these species were clearly separated from the remaining species (*P*. *canadensis* and *P*. *deltoides × P*. *nigra* cv. Chile are hybrids from crosses between *P*. *deltoides* and *P*. *nigra*, and *P*. *deltoides ‘Shan Hai Guan’* and *P*. *deltoides ‘Lux’* are cultivars of *P*. *deltoides*) belonging to the *P*. *deltoides* maternal lineage and clustered with species in section *Leuce*, whereas they were not identified using the *ITS* phylogeny. *P*. *fremontii* had been a subspecies of *P*. *deltoides* until Flora of North America considered it a separate species. Our chloroplast phylogeny analysis supported its high maternal homology with *P*. *deltoides*.

The diversity of *Tacamahaca* species and their distribution areas is very suitable for analyzing the phylogeny of *Populus*[11]. However, most species are wild types and are difficult to collect. This limits the phylogenetic reconstruction of section *Tacamahaca* and even the genus *Populus*. Previous research has shown the complexity in origin and evolution that, in most cases, has led to large genetic distances between consectional species[11–12, 17, 50]. This section is thus thought to be paraphyletic, and the interspecific relationship is most complicated. Our study supports the paraphyletic nature of section *Tacamahaca* after analyzing 24 species or hybrids. The results show high interspecific pairwise distance values and error bars for both the chloroplast and *ITS* datasets, which indicate distinct genetic differences among these species. Section *Tacamahaca* species were divided into two clades (1 and 2) in the chloroplast Bayes tree, in which the species in subclades 1 and 4 had overlapping distribution, suggesting its two lineages. These lineages showed frequent gene flow, reflecting nuclear genome affinity with recombination during concerted evolution, explaining why taxonomic positions differed between the chloroplast and *ITS* phylogenic trees.

Cervera *et al*.[12] found that section *Leucoides* showed interspecific heterogeneous relationships. The four species in section *Leucoides* from this study, namely, *P*. *lasiocarpa*, *P*. *wilsonii*, *P*. *gonggaensis* and *P*. *pseudoglauca*, also produced high pairwise distance values and had different phylogenetic positions (especially for *P*. *gonggaensis*). This indicated a paraphyletic nature, although it is doubtful whether *P*. *gonggaensis* can be considered a separate species.

### The phylogenetic origin and evolution of *Populus*

It has been conclusively confirmed by many studies that *Populus* is of monophyletic origin[9, 11–12, 51]. During the subsequent reticulate evolution, the genesis of new species or speciation has brought about the diversification of lineages, which are widely accepted to divided into six sections at present. Phylogenetic analyses, especially those based on gene sequences, are one of the most important and widely used ways to reconstruct the evolutionary process[52–53]. Phylogenetic analysis based on AFLP[12] and *ITS*[51] data showed that section *Leuce* was the most basal lineage in the genus *Populus*. *ITS* sequence-based phylogeny from this study also defined it as a basal taxon of the tree.

However, the opinion of section *Leuce* as the basal lineage contradicts the fossil records. Fossils are the only unequivocal proof of the actual relationships between leaves, stems and reproductive organs[54]. *P*. *wilmattae*, one of the earliest probable *Populus* fossil species known, is remarkably similar to the extant species *P*. *mexicana* from section *Abaso*[6, 54]. *P*. *mexicana* had been placed in section *Aigeiros* until Eckenwalder[55] made the taxonomic decision to place it in a new section, “*Abaso*”, after unscrambling the morphological, distributional, ecological and paleobotanical information. Further analysis based on morphological evidence showed that *P*. *mexicana* was closely related to section *Turanga*, followed by section *Aigeiros*[4, 56]. Our chloroplast data clustered section *Abaso*, *Aigeiros* and *Turanga* into clade 1 in the Bayes tree. Consequently, this clade characterizes more traits of earliest probable fossil species *P*. *wilmattae* than clade 2, including section *Leuce*. The appearance of section *Leuce* as the basal taxon of the *ITS* tree is related to the fact that it might have little reticulate evolution with other sections and was clustered into a species-poor group. A widespread misunderstanding occurs when researchers consider species-poor groups as basal branches and interpret them as ancestral [57–59].

Moreover, species within clade 1 (involving five sections except for *Leuce*) clustered coinciding with their specific geographical distribution areas. Species within subclade 1 were geographically restricted mainly in China (except for *P*. *ilicifolia*, located in East Africa), while subclade 2 only contained North America species. These findings suggested that geographical isolation is a main factor contributing to diversification of *Populus* lineages and that convergent evolution of chloroplast may function in their evolutionary process.

After comparing the morphological characteristics, section *Leucoides* was found to be similar to section *Turanga*. In other words, section *Leucoides* might be an ancestral member of *Populus*. In addition, its preference for permanent swamp accords with the hypothesis that the *Populus* ancestor is a mountain species[4, 60]. Our chloroplast phylogenetic tree showed that, although it contained only four species, section *Leucoides* was closely linked to section *Tacamahaca*. *P*. *gonggaensis* and *P*. *wilsonii* clustered with the subclade 1 species (e.g., *P*. *cathayana*) in section *Tacamahaca*, whereas *P*. *lasiocarpa* and *P*. *pseudoglauca* clustered with the subclade 4 species (e.g., *P*. *simonii*) in section *Tacamahaca*. However, phylogenetic analysis did not conclusively delimitate their section boundaries.

### The phylogenetic evolution of *P*. *szechuanica* var. *tibetica*

Probable *Populus* fossils have been found that date from the Upper Cretaceous to the Oligocene ages[61–62]. Without extant (ignoring introduced members of northern taxa) and fossil species from the Southern Hemisphere, Raven and Axelrod[63] suggested that *Populus* was in Laurasian but did not confirm the specific location. The abundant genetic resources for *Populus* and the geological history of Southwest China suggested that this region might be a center of the genus *Populus*[64–67]. Gong[60] also supported the hypothesis that *Populus* originated from Southwest China after combining data from fossil, paleogeographic, paleoclimatic, and geographic information sources, etc.

Species in phylogenetic trees grouped generally along their species lines. However, *P*. *szechuanica and P*. *szechuanica* var. *tibetica* in Southwest China were found to be exceptions. For *P*. *szechuanica*, two specimens we collected were located in subclade 4 of the chloroplast Bayes tree, and one specimen (MG262357) obtained from GenBank was distributed to subclade 1. Because we lack information for MG262357, we do not know whether the phenomenon reflects a real difference within species or just a specimen misidentification.

*P*. *szechuanica* var. *tibetica* is a variety of *P*. *szechuanica* according to classical taxonomy and is widely distributed at altitudes of approximately 2000–4500 m above sea level in the Tibet Plateau and adjacent areas[68–69]. The study based on EST-SSR[69] revealed that low genetic differentiation was attributable to populations with genotypes from low-, medium- and high-altitude species in the Sejila Mountain area, and there was no clear correlation with altitude. SSR analysis performed by Bo[70] divided four natural populations of Tibetan poplar into two groups. One group contained populations from Nyingchi and Lhasa, and the other contained populations from Xigaze and Shannan. Variations were found mainly within individuals, and no significant correlation was found between genetic and geographical distances. After analysis of taxonomic position by *trnL-F* sequence, Wei *et al*.[42] found that *P*. *szechuanica* var. *tibetica* was independent of the other *Populus* species used in his study, which suggested an independent evolutionary path that correlated with willow (*Salix*).

**Fig 7:**
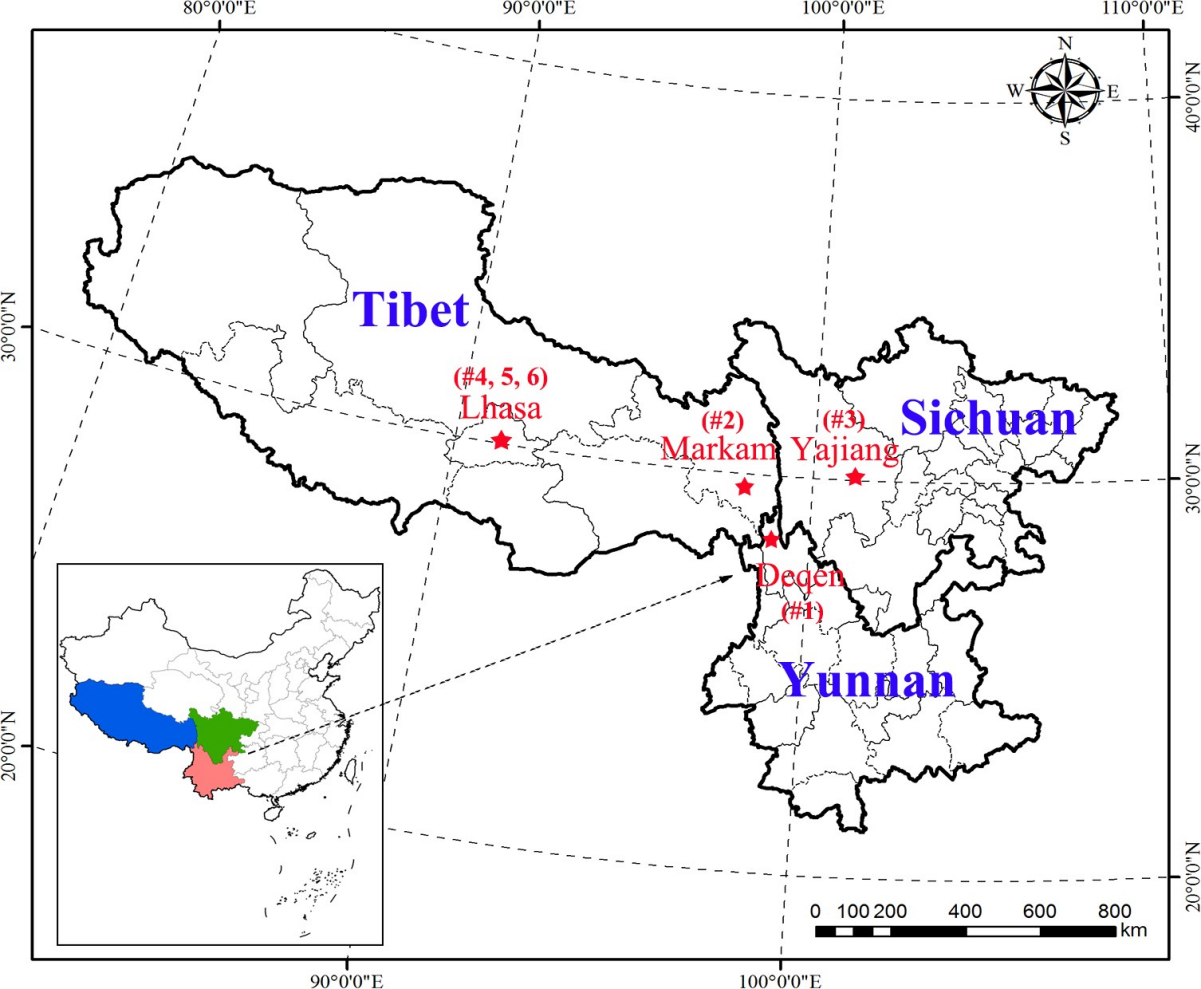
A map of Southwest China showing the collection points for *P*. *szechuanica* var. *tibetica* specimens. Solid stars represent the sample collection positions. A total of five positions belonging to three geographical regions were used in this study. Numbers inside parentheses represent the code for the specimens collected at this point.

The phylogeny in this study showed that, over a wider region, specimens of *P*. *szechuanica* var. *tibetica* clustered coinciding with their geographical location. Three specimens (#4, 5 and 6) of *P*. *szechuanica* var. *tibetica* from Lhasa grouped with subclade 1 (Fig 4) species, such as *P*. *cathayana*, *P*. *wilsonii* and *P*. *euphratica*, based on the chloroplast Bayes tree and were located as the basal taxa of the *ITS* trees. The natural barriers, i.e. the Tibetan Plateau and Hengduan Mountains, had made a relatively enclosed space and prevented gene flow with conspecific and consectional *Populus*. In contrast, the two specimens of *P*. *szechuanica* var. *tibetica* from Deqin (#1) and Yajiang (#3) were separated from three specimens in Lhasa by both chloroplast and *ITS* analyses and were always near the top of the tree. The #2 sample of *P*. *szechuanica* var. *tibetica* from Markam was a transitional type because it clustered with specimens #4, 5 and 6 from Lhasa based on chloroplast phylogeny and with specimens #1 from Deqin and #3 from Yajiang based on *ITS* phylogeny. The combination of phylogenetic position and geographical region seemed to provide an evolutionary path (Fig 7), but further detailed studies with more populations and specimens are needed to confirm this possibility.

In conclusion, our study has focused on the phylogenetic relationships of *Populus* and has revealed the intrasectional relationships and reticulate evolutional patterns, which confirmed some of the hypotheses put forward in previous studies and offers some new suggestions. Multiple gene trees and extensive geographical species are effective resources when assessing the systematics and reconstructing the phylogeny of *Populus*. However, further analyses on more specimens (e.g., *P*. *szechuanica* var. *tibetica* population), species (e.g., *P*. *mexicana*) and sequence information (e.g., single-copy nuclear genes and all chloroplast genomes) are required.

## Supporting information

S1 Table Source of *Populus*. (XLSX)

S2 Table List of primers used in this study. (DOCX)

## References

[1] Wilde HD, Meagher RB, Merkle SA. Expression of foreign genes in transgenic yellow-poplar plants. Plant Physiology. 1992; 98(1): 114–120. 10.1104/pp.98.1.114 16668600 PMC1080157

[2] Ding CJ, Liang LX, Diao S. Genome-wide analysis of day/night DNA methylation differences in *Populus nigra*. PLoS One. 2018; 13(1): e0190299 10.1371/journal.pone.0190299 29293569 PMC5749751

[3] Ding LP, Chen YJ, Wei XL, Ni M, Zhang JW, Wang HZ, et al Laboratory evaluation of transgenic *Populus davidiana* × *Populus bolleana* expressing *Cry1Ac*+*SCK*, *Cry1Ah3*, and *Cry9Aa3* genes against gypsy moth and fall webworm. PLoS One. 2017; 12(6): e0178754 10.1371/journal.pone.0178754 28582405 PMC5459438

[4] Eckenwalder JE. Systematics and evolution of *Populus* In: Stettler RF, Bradshaw HD, Heilman PE, Hinckley TM, editors. Biology of *Populus* and its implications for management and conservation. Montreal, Canada: NRC research Press; 1996 pp. 7–32.

[5] Fang ZF, Zhao SD, Skvortsov AK. Salicaceae Mirbel: 1. *Populus* Linnaeus In: Wu CY, Raven PH, editors. Flora of China Vol 4 Beijing: Science Press & St. Louis: Missouri Botanical Garden Press; 1999 pp. 139–163.

[6] Difazio SP, Slavov GT, Joshi CP. *Populus*: a premier pioneer system for plant genomics In: Joshi CP, Difazio SP, Kole C, editors. Genetics, genomics and breeding of poplar. Lebanon: Science Publishers; 2011 pp. 1–28.

[7] Ma CG. Researches on poplar breeding in China to be viewed in the light of the development and achievement of cross breeding of poplars in the world. World Forestry Research. 1994; 3: 23–30.

[8] Taylor G. *Populus*: arabidopsis for forestry. Do we need a model tree? Annals of Botany. 2002; 90(6): 681–689. 10.1093/aob/mcf255 12451023 PMC4240366

[9] Hamzeh M, Dayanandan S. Phylogeny of *Populus* (Salicaceae) based on nucleotide sequences of chloroplast *trnT*-*trnF* region and nuclear rDNA. American Journal of Botany. 2004; 91(9): 1398–1408. 10.3732/ajb.91.9.1398 21652373

[10] Hamzeh M, Perinet P, Daanandan. Genetic relationships among species of *Populus* (Salicaceae) based on nuclear genomic data. The Journal of Torrey Botanical Society. 2006; 133(4): 519–527. 10.3159/1095-5674(2006)133[519:GRASOP]2.0.CO;2

[11] Wang ZS, Du SH, Dayanandan S, Wang DS, Zeng YF, Zhang JG. Phylogeny reconstruction and hybrid analysis of *Populus* (Salicaceae) based on Nucleotide sequences of multiple single-copy nuclear genes and plastid fragments. PloS One. 2014; 9(8): e103645 10.1371/journal.pone.0103645 25116432 PMC4130529

[12] Cervera MT, Storme V, Soto A, Ivens B, Van Montagu M, Rajora OP, et al Intraspecific and interspecific genetic and phylogenetic relationships in the genus *Populus* based on AFLP markers. Theoretical and Applied Genetics. 2005; 111(7): 1140–1456. 10.1007/s00122-005-0076-2 16211377

[13] Page RDM, Charleston MA. From gene to organismal phylogeny: reconciled trees and the gene tree/species tree problem. Molecular Phylogenetics and Evolution. 1997; 7(2): 231–240. 10.1006/mpev.1996.0390 9126565

[14] Burbrink FT, Pyron RA. The impact of gene-tree/species-tree discordance on diversification-rate estimation. Evolution. 2011; 65(7): 1851–1861. 10.1111/j.1558-5646.2011.01260.x 21729043

[15] Dondi R, Ei-Mabrouk N, Swenson KM. Gene tree correction for reconciliation and species tree inference: complexity and algorithms. Journal of Discrete Algorithms. 2014; 25: 51–65. doi: 10.1016/j.jda. 2013.06.001 10.1186/1748-7188-7-31 PMC3567949 23167951

[16] Degnan JH, Rosenberg NA. Gene tree discordance, phylogenetic inference and the multispecies coalescent. Trends in Ecology & Evolution. 2009; 24(6): 332–340. 10.1016/j.tree.2009.01.009 19307040

[17] Feng JJ, Jiang DC, Shang HY, Dong M, Wang GN, He XY, et al Barcoding poplars (*Populus* L.) from western China. PloS One. 2013; 8(8): e71710 10.1371/journal.pone.0071710 23977122 PMC3747233

[18] Ferreri M, Qu W, Han B. Phylogenetic networks: a tool to display character conflict and demographic history. African Journal of Biotechnology. 2011; 10(60): 12799–127803. 10.5897/AJB11.010

[19] Huson DH, Scornavacca C. A survey of combinatorial methods for phylogenetic networks. Genome Biology and Evolution. 2011; 3: 23–35. 10.1093/gbe/evq077 21081312 PMC3017387

[20] Mort ME, Archibald JK, Randle CP, Levsen ND, O'Leary TR, Topalov K, et al Inferring phylogeny at low taxonomic levels: utility of rapidly evolving cpDNA and nuclear *ITS* loci. American Journal of Botany. 2007; 94(2): 173–183. 10.3732/ajb.94.2.173 21642219

[21] Bargues MD, Zuriaga MA, Mas-Coma S. Nuclear rDNA pseudogenes in Chagas disease vectors: evolutionary implications of a new 5.8S+ITS-2 paralogous sequence marker in triatomines of North, Central and northern South America. Infection Genetics and Evolution. 2014; 21: 134–156. 10.1016/j.meegid.2013.10.028 24239656

[22] Kress WJ, Erickson DL. A two-locus global DNA barcode for land plants: the coding *rbcL* gene complements the non-coding *trnH-psbA* spacer region. PloS One. 2007; 2(6): e508 10.1371/journal.pone.0000508 17551588 PMC1876818

[23] Von Crautlein M, Korpelainen H, Pietilainen M, Rikkinen J (2011) DNA barcoding: a tool for improved taxon identification and detection of species diversity. Biodiversity and Conservation 20: 373–389. 10.1007/s10531-010-9964-0

[24] Chase MW, Cowan RS, Hollingsworth PM, Van Den Berg C, Madrinan S, Petersen G, et al A proposal for a standardised protocol to barcode all land plants. Taxon. 2007; 56(2): 295–299.

[25] Lahaye R, Van Der Bank M, Bogarin D, Warner J, Pupulin F, Gigot G, et al DNA barcoding the floras of biodiversity hotspots. Proceedings of the National Academy of Sciences. 2008; 105(8): 2923–2928. 10.1073/pnas.0709936105 18258745 PMC2268561

[26] Lahaye R, Savolainen V, Duthoit S, Maurin O, Van Der Bank M. A test of *psbK-psbI* and *atpF-atpH* as potential plant DNA barcodes using the flora of the Kruger National Park as a model system (South Africa). Available from Nature Precedings http://hdl.handle.net/10101/npre.2008.1896.1. 2008.

[27] Taberlet P, Gielly L, Pautou G, Bouvet J. Universal primers for amplification of three non-coding regions of chloroplast DNA. Plant Molecular Biology. 1991; 17(5): 1105–1109. 10.1007/BF00037152 1932684

[28] White TJ, Bruns T, Lee S, Taylor JW. Amplification and direct sequencing of fungal ribosomal RNA genes for phylogenetics In: Innis MA, Gelfand DH, Sninsky JJ, White TJ, editors. In PCR protocols: a guide to methods and applications. San Diego: Academic Press; 1990 pp. 315–322.

[29] Murray MG, Thompson WF. Rapid isolation of high molecular weigh plant DNA. Nucleic Acids Research. 1980; 8(19): 4321–4326. 10.1093/nar/8.19.4321 7433111 PMC324241

[30] Thompson JD, Gibson TJ, Plewniak F, Jeanmougin F, Higgins DG. The CLUSTAL X windows interface: flexible strategies for multiple sequence alignment aided by quality analysis tools. Nucleic Acids Research. 1997; 25(24): 4876–4882. 10.1093/nar/25.24.4876 9396791 PMC147148

[31] Tamura K, Peterson D, Peterson N, Stecher G, Nei M, Kumar S. MEGA5: molecular evolutionary genetics analysis using maximum likelihood, evolutionary distance, and maximum parsimony methods. Molecular Biology and Evolution. 2011; 28(10): 2731–2739. 10.1093/molbev/msr121 21546353 PMC3203626

[32] Farris JS. Kallersjo M, Kluge AG, Bult C. Testing significance of incongruence. Cladistics, 1995; 10(3): 315–319. 10.1111/j.1096-0031.1994.tb00181.x

[33] Swofford DL. PAUP* Phylogenetic analysis using parsimony (* and other methods). Version 4.0. Sunderland, Massachusetts: Sinauer Associates; 2002.

[34] Felsenstein J. Confidence limits on phylogenies: an approach using the bootstrap. Evolution. 1985; 39(4): 783–791. 10.1111/j.1558-5646.1985.tb00420.x 28561359

[35] Ronquist F, Huelsenbeck JP. MrBayes 3: Bayesian phylogenetic inference under mixed models. Bioinformatics. 2003; 19: 1572–1574. 10.1093/bioinformatics/btg180 12912839

[36] Posada D, Crandall KA. Modeltest: testing the model of DNA substitution. Bioinformatics. 1998; 14(9): 817–818. 10.1093/bioinformatics/14.9.817 9918953

[37] Som A., Causes consequences and solutions of phylogenetic incongruence. Briefings in Bioinformatics. 2015; 16(3): 536–548. 10.1093/bib/bbu015 24872401

[38] Jeffroy O, Brinkmann H, Delsuc F, Philippe H. Phylogenomics: the beginning of incongruence? Trends in Genetics. 2006; 22(4): 225–231. 10.1016/j.tig.2006.02.003 16490279

[39] Leple JC, Pilate G, Jouanin L. Transgenic poplar trees (*Populus* species). Biotechnology in Agriculture and Forestry. 2000; 44: 221–244.

[40] Fu MF, Fan JF, Zhou YX, Gao JS. Relationship among species of sect. *Populus* using RAPD markers. Acta Botanica Boreali-Occidentalia Sinica. 2009; 29(12): 2408–2414.

[41] Cicatelli A, Sala F, Yfan H, Lupi R. Genetic biodiversity and phylogenetic studies in poplar by means of the metallothionein multigene family. The Molecular Basis of Plant Genetic Diversity. 2012; 113–134.

[42] Wei ZZ, Guo LQ, Zhang JF, Li BL, Zhang DQ, Guo H. Phylogenetic relationship of *Populus* by *trnL-F* sequence analysis. Journal of Beijing Forestry University. 2010; 32(2): 27–33.

[43] Li KY, Huang MR, Wang MX, Chen D, He ZX. Study on DNA polymorphisms and phylogenetics of *Populus*: *Aigeiros*, *Tacamahaca* and *Leuce* section. Journal of Nanjing Forestry University. 1996; 20(1): 6–11.

[44] Li SW, Zhang YH, Zhang ZY, An XM, He CZ, Li BL. AFLP analysis of some species and hybrids in *Populus*. Scientia Silvae Sinicae. 2007; 43(1): 37–41.

[45] Rajora OP, Dancik BP. Chloroplast DNA variation in *Populus*. I. Intraspecific restriction fragment diversity within *Populus deltodides*, *P*. *nigra*, and *P*. *maximowiczii*. Theoretical and Applied Genetics. 1995; 90(3–4): 317–323. 10.1007/BF00221971 24173919

[46] Rajora OP, Dancik BP. Chloroplast DNA variation in *Populus*. II. Intraspecific restriction fragment polymorphisms and genetic relationships among *Populus deltodides*, *P*. *nigra*, *P*. *maximowiczii* and *P*. *× canadensis*. Theoretical and Applied Genetics. 1995; 90(3–4): 324–330. 10.1007/BF00221972 24173920

[47] Rajora OP, Dancik BP. Chloroplast DNA variation in *Populus*. III. Novel chloroplast DNA variants in natural *Populus × canadensis* hyirds. Theoretical and Applied Genetics. 1995 90(3–4): 331–334. 10.1007/BF00221973 24173921

[48] Racchi ML, Turchi A, Caparrini S, Camussi A. SSCP intron marker system is a convenient tool for clonal fingerprinting of poplar (*Populus*) cultivars of different species and interspecific hybrids. Genetic Resources and Crop Evolution. 2011; 58(4): 507–518. 10.1007/s10722-010-9594-0

[49] Smith RL, Sytsma KJ. Evolution of *Populus nigra* (sect. *Aigeiros*): introgressive hybridization and the chloroplast contribution of *Populus alba* (sect. *Populus*). America journal of botany. 1990; 77(9): 1176–1187. 10.2307/2444628

[50] Liu X, Wang ZS, Wang DS, Zhang JG. Phylogeny of *Populus*-*Salix* (Salicaceae) and their relative genera using molecular datasets. Biochemical Systematics and Ecology. 2016, 210–215. 10.1016/j.bse.2016.07.016

[51] Shi QL, Zhu GQ, Huang MR, Wang MX. Phylogenetic relationship of *Populus* sections by *ITS* sequence analysis. Acta Botanica Sinica. 2001; 43(3): 323–325.

[52] De Bruyn A, Martin DP, Lefeuvre P (2014) Phylogenetic reconstruction methods: an overview. Molecular Plant Taxonomy 1115, 257–277. 10.1007/978-1-62703-767-9_13 24415479

[53] Higashi H, Ikeda H, Setoguchi H. Molecular phylogeny of *Shortia sensulato* (Diapensiaceae) based on multiple nuclear sequences. Plant Systematics and Evolution. 2015; 301(2): 523–529. 10.1007/s0060

[54] Manchester SR, Dilcher DL, Tidwell WD. Interconnected reproductive and vegetative remains of *Populus* (Salicaceae) from the middle Eocene Green River Formation, northeastern Utah. American Journal of Botany. 1986; 73(1): 156–160. 10.1002/j.1537-2197.1986.tb09691.x 30139119

[55] Eckenwalder JE. North American cottonwoods (*Populus*, Salicaceae) of sections *Abaso* and *Aigeiros*. Journal of the Arnold Arboretum. 1977; 58(3): 193–208.

[56] Zhao N, Gong GT, Liu J. On the taxonomic position of *Populus mexicana* wesmael in north America. Journal of Sichuan Forestry Science and Technology. 1997; 18(2): 1–5.

[57] Krell F, Cranston FS. Which side of the tree is more basal? Systematic Entomology. 2004; 29(3): 279–281. 10.1111/j.0307-6970.2004.00262.x

[58] Crisp MD, Cook LG. Do early branching lineages signify ancestral traits? Trends in Ecology & Evolution. 2005; 20(3): 122–128. 10.1016/j.tree.2004.11.010 16701355

[59] Omland KE, Cook LG, Crisp MD. Tree thinking for all biology: the problem with reading phylogenies as ladders of progress. BioEssays. 2008; 30(9): 854–867. 10.1002/bies.20794 18693264

[60] Gong GT. The geographic distribution and origin of *Populus* L. Journal of Sichuan Forestry Science and Technology. 2004; 25(2): 25–30.

[61] Muller J. Fossil pollen records of extant angiosperms. The Botanical Review. 1981; 47(1): 1–142. 10.1007/BF02860537

[62] Taylor DW. Paleobiogeographic relationships of angiosperms from the Cretaceous and early Tertiary of the north American area. The Botanical Review. 1990; 56(4): 279–417. 10.1007/BF02995927

[63] Raven PH, Axelrod DI. Angiosperm biogeography and past continental movements. Annals of the Missouri Botanical Garden. 1974; 61(3): 539–657. 10.2307/2395021

[64] Wan XQ, Zhang F, Zhong Y, Ding YH, Wang CL, Hu TX. Study of genetic relationships and phylogeny of the native *Populus* in southwest China based on nucleotide sequences of chloroplast *trnT*-*trnF* and nuclear DNA. Plant Systematics and Evolution. 2013; 299(1): 57–65. 10.1007/s00606-012-0702-9

[65] Xu WY. Poplar Harbin, Heilongjiang: People’s Press; 1988.

[66] Luan HH, Su XH, Zhang BY. Research progress in genetic evaluation of *Populus* L. germplasm resources. Chinese Bulletin of Botany. 2011; 46(5): 586–595.

[67] Wan XQ, Zhang F. An overview of *Populus* genetic resources in southwest China. The Forestry Chronicle. 2013; 89(1): 79–87. 10.5558/tfc2013-013

[68] Tang YD, Pubu C, Cidan Z. Biological characteristics of *Populus szechuanica* var. *tibetica*, the rare and endemic plant of Qinghai-Tibetan Plateau in the different local environment. Chinese Wild Plant Resources. 2012; 31(2): 24–28, 32.

[69] Shen DF, Bo WH, Xu F, Wu RL. Genetic diversity and population structure of the Tibetan poplar (*Populus szechuanica* var. *tibetica*) along an altitude gradient. BMC Genetics. 2014; 15 (1): S11 10.1186/1471-2156-15-S1-S11 25079034 PMC4118629

[70] Bo WH. The genetic diversity and hybrid filial variation study in *Populus szechuanica* var. *tibetica*. Doctor thesis, Beijing Forestry University. 2012.

