# Supplementary material for: Analyzing the phylogeny of poplars based on molecular data
Source: PLoS One. 2018 Nov 9;13(11):e0206998. doi: 10.1371/journal.pone.0206998 (PMC6226168; doi:10.1371/journal.pone.0206998)
Supplement: S2 Table — (DOCX) [file pone.0206998.s002.docx]

**Table S2.** **List of primers used in this study**

| Region | Direction | Primer sequences (5′−3′) | Anneal / °C | Reference |
| --- | --- | --- | --- | --- |
| *rbcL-a* | *F* | ATGTCACCACAAACAGAGACTAAAGC | 57 | [22] |
|  | *R* | GTAAAATCAAGTCCACCRCG |  |  |
| *matK* | *f* | CATCTAGAAAAATTGGTTCA | 54 | [23] |
|  | *r* | TTTAACACAAGAAAGTCGAAG |  |  |
| *rpoB* | *1f* | AAGTGCATTGTTGGAACTGG | 59 | [24] |
|  | *4r* | GATCCCAGCATCACAATTCC |  |  |
| *ropC1* | *2f* | GGCAAAGAGGGAAGATTTCG | 59 | [24] |
|  | *4r* | CCATAAGCATATCTTGAGTTGG |  |  |
| *psbA-trnH* | *psbA* | GTTATGCATGAACGTAATGCTC | 57 | [25] |
|  | *trnH* | CGCGCATGGTGGATTCACAATCC |  |  |
| *psbI-psbK* | *psbI* | AGAGTTTGAGAGTAAGCAT | 57 | [26] |
|  | *psbK* | TTAGCCTTTGTTTGGCAAG |  |  |
| *atpF-atpH* | *atpF* | ACTCGCACACACTCCCTTTCC | 57 | [26] |
|  | *atpH* | GCTTTTATGGAAGCTTTAACAAT |  |  |
| *trnL-F* | *trnL* | CGAAATCGGTAGACGCTACG | 59 | [27] |
|  | *trnF* | ATTTGAACTGGTGACACGAG |  |  |
| *ITS* | *ITS1* | TCCGTAGGTGAACCTGCGC | 57 | [28] |
|  | *ITS4* | TCCTCCGCTTATTGATATGC |  |  |
